# Pharmacological effects, molecular mechanisms and strategies to improve bioavailability of curcumin in the treatment of neurodegenerative diseases

**DOI:** 10.3389/fphar.2025.1625821

**Published:** 2025-07-10

**Authors:** Gang Wang, Xueyuan Zhou, Xiaoyan Pang, Ke Ma, Lu Li, Yuexin Song, Dongxia Hou, Xiaohua Wang

**Affiliations:** ^1^ Department of Genetics, Inner Mongolia Maternity and Child Health Care Hospital, Hohhot, China; ^2^ Inner Mongolia Engineering Research Center of Medical Genetics, Hohhot, China

**Keywords:** curcumin, natural medicine, neurodegenerative disease, pharmacological activity, bioavailability

## Abstract

With the global population aging, the incidence of neurodegenerative diseases (NDs), such as Alzheimer’s disease, Parkinson’s disease, Huntington’s disease and amyotrophic lateral sclerosis, has been progressively increasing. However, effective therapeutic strategies and clinical drugs for these disorders remain scarce. Curcumin, a natural polyphenolic compound primarily derived from the herbaceous plant *Curcuma longa* L., has been proposed as a promising candidate for ND treatment based on the excellent antioxidant, anti-inflammatory and neuroprotective properties. Its pharmacological activities encompass scavenging reactive oxygen species, mitigating toxic protein aggregation and cytotoxicity, repairing mitochondrial dysfunction, and inhibiting excessive neuronal apoptosis. Compared with synthetic drugs, curcumin demonstrates a more favorable safety profile with fewer side effects. Nevertheless, its clinical application is substantially hindered by poor bioavailability, which stems from low aqueous solubility, inefficient intestinal absorption, and rapid metabolism and systemic elimination. Conventional administration methods often fail to achieve effective concentrations *in vivo*. Further clinical trials are also required to validate the therapeutic efficacy and potential adverse effects in human subjects. This article systematically reviews the pathogenesis of NDs and the knowledge on curcumin including pharmacological effects, neuroprotective mechanisms, functions across specific NDs and advanced strategies to enhance the bioavailability, with the aim of promoting the development and clinical translation of curcumin-based therapeutics for NDs.

## 1 Introduction

Neurodegenerative diseases (NDs) represent a group of disorders characterized by progressive degeneration of the structure and function of the nervous system, particularly affecting neurons in the brain and spinal cord. These conditions often arise from a complex interplay of genetic, environmental and lifestyle factors (Zhang W. et al., 2023; Swaddiwudhipong et al., 2023). The most prevalent NDs include Alzheimer’s disease (AD), Parkinson’s disease (PD), amyotrophic lateral sclerosis (ALS) and Huntington’s disease (HD). Typical symptoms encompass memory loss, cognitive decline, motor dysfunction and behavioral changes, which vary depending on the specific disease and affected brain regions. The pathogenesis involves multiple interconnected mechanisms, including genetic mutations, protein misfolding and aggregation, oxidative stress, mitochondrial dysfunction and chronic neuroinflammation (Zhao et al., 2020; Song et al., 2020). NDs are closely related to age, as the onset risk increases and symptom severity worsens with advancing age (Sun et al., 2024). Consequently, the global aging of the population is expected to increase the incidence and prevalence of NDs, presenting a major challenge for healthcare systems worldwide (Pichet Binette et al., 2020; Shen et al., 2021). Current therapeutic strategies predominantly focus on symptom management and slowing disease progression. For instance, cholinesterase inhibitors and NMDA receptor antagonists are widely used for AD, while dopamine replacement therapies remain the standard for PD (D'Angremont et al., 2023; Liang et al., 2024). However, these treatments are not curative and only provide temporary relief. Long-term use may lead to reduced efficacy and increased adverse effects (Xu et al., 2023). In recent years, significant advancements has been made in drug development, with a growing emphasis on targeting the underlying pathological mechanisms. Key targets include β-amyloid (Aβ) and tau proteins of AD, α-synuclein (α-syn) of PD and huntingtin proteins of HD (Ran and Zhu, 2024; Martic, 2022). Additionally, emerging approaches like gene therapy, immunotherapies and stem cell-based treatments also hold great potential, although many are still in clinical trial stages (Sun and Roy, 2021). The development of more effective interventions and novel drugs is urgently required to address the multifaceted challenges posed by NDs.

**FIGURE 1:**
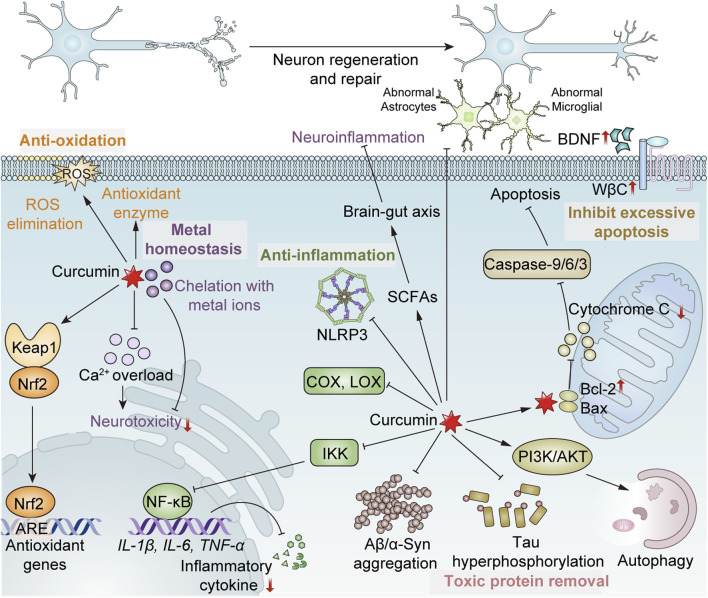
Mechanism of curcumin exerting the neuroprotective effect. (1) Antioxidation: Ⅰ. Direct scavenging of ROS; Ⅱ. Enhancement of antioxidant enzyme activity, such as SOD, CAT and GPx; Ⅲ. Promoting the dissociation of Nrf2 from Keap1 to bind to antioxidant response elements (ARE), inducing the expression of antioxidant genes. (2) Anti-inflammation: Ⅰ. Inhibition of the activities of cyclooxygenase (COX), lipoxygenase (LOX), etc.; Ⅱ. Inhibition of the activation of the inflammasome NLRP3; Ⅲ. Inhibiting the activity of the IκB kinase (IKK) complex, thereby suppressing NF-κB, and consequently reducing the expression of NF-κB-mediated pro-inflammatory factors such as IL-1β, IL-6, TNF-α, etc.; Ⅳ. Reduced abnormal activation of astrocytes and microglia; Ⅴ. Promoting the production of short-chain fatty acids (SCFAs) of intestinal microbes to regulate the brain-gut axis, maintaining the integrity of the blood-brain barrier and indirectly protecting the nervous system. (3) Removal of toxic proteins: Ⅰ. Inhibition of the formation of toxic protein aggregates; Ⅱ. Prevention of tau phosphorylation; Ⅲ. Activating the PI3K/AKT/mTOR pathway to promote autophagic clearance of formed toxic proteins and damaged cells. (4) Maintenance of metal homeostasis: Ⅰ. Chelating heavy metal ions to alleviate neurotoxicity; Ⅱ. Prevention of intracellular calcium overload. (5) Inhibition of excessive apoptosis: Upregulating Bcl-2 and downregulating Bax to reduce mitochondrial cytochrome C release and inhibit caspase-9/6/3 activation, suppressing excessive apoptosis. (6) Promotion of regeneration and repair of neurons: Ⅰ. Increasing the expression of brain-derived neurotrophic factor (BDNF); Ⅱ. Upregulation of Wnt/β-catenin (WβC) pathway.

Curcumin, a natural polyphenolic compound, is primarily derived from the rhizomes of *Curcuma longa* L. (turmeric) which is a perennial herbaceous plant that has been extensively utilized in traditional medicine and culinary practices (Rafiee et al., 2019). The synthesis of curcumin has been also investigated to develop optimal procedures for the rapid acquisition (Ahmed et al., 2017). The chemical structure is characterized by two aromatic rings linked by a seven-carbon chain with β-diketone functional groups, conferring the diverse biological activities (Figure 1). Curcumin exhibits a broad spectrum of pharmacological properties, including anti-inflammatory, antioxidant, anti-cancer and neuroprotective effects, making it a promising therapeutic candidate for NDs (Tandon et al., 2020). It can inhibit the aggregation of pathological proteins, mitigate oxidative stress and suppresses neuroinflammation. Furthermore, curcumin enhances the expression of neurotrophic factors and promotes autophagy, aiding in the clearance of damaged cellular components (Gao et al., 2020; Namgyal et al., 2021). Despite its prospective pharmaceutical benefits, the clinical application of curcumin is hampered by poor bioavailability due to low solubility, structural instability, rapid metabolism and fast systemic elimination. These pharmacokinetic challenges prevent it from reaching an effective concentration in target tissues. To overcome the limitations, some strategies have been proposed such as the development of curcumin analogs, nanoparticle-based delivery systems, liposomal encapsulation and the use of adjuvants like piperine (Olmos-Juste et al., 2021; Liu et al., 2020; Zhang et al., 2018). It was demonstrated that curcumin nano-formulations exhibited excellent solubility and stability, along with antioxidant and enzyme inhibition properties, as evidenced by molecular docking and simulation studies (Kanwal et al., 2023). These approaches are of significant potential in improving the efficacy and feasibility of curcumin in the treatment of neurodegenerative disorders.

In light of the increasing interest in natural medicine for the management of NDs, curcumin has garnered extensive attention as a highly promising therapeutic agent. Some clinical studies have explored the neuroprotective effects of curcumin, but their overall findings remain promising yet inconclusive (Table 1). Most trials were short-term and involved small sample sizes, with considerable dosage variability. Particularly noteworthy is the heterogeneity in therapeutic outcomes among studies targeting the same neurodegenerative conditions, such as AD. Trials employing unmodified prototype curcumin preparations showed negligible differences from placebo controls, primarily due to the inherently low bioavailability and inadequate systemic absorption. This review systematically synthesizes the knowledge on curcumin, including the pharmacological effects, underlying neuroprotective mechanisms, functions across specific NDs, and cutting-edge strategies for bioavailability enhancement. Meanwhile, the limitations of current research and the unresolved issues requiring further investigation are critically discussed. These insights will contribute to the advancement of novel drug development and the translation of curcumin-based therapies toward clinical practice.

**TABLE 1:** Clinical trials of curcumin on the effect of central nervous system.

No.	Object	Research type	Formulation/Dosage	Group	Duration	Outcome	Side effect	References
1	40 non-demented adults	Randomized, double-blind, and placebo-controlled	Highly absorptive curcumin dispersed with colloidal nanoparticles-Theracurmin, 90 mg twice daily (180 mg/day)	Placebo-19; Intervention-21	18 months	Memory and attention improved with Theracurmin; less neuropathological accumulation in the amygdala and hypothalamus	Four curcumin-treated and two placebo-treated subjects experienced gastrointestinal side effects (transient abdominal pain, gastritis, or nausea)	Small et al. (2018)
2	36 patients with mild-to-moderate AD	Randomized, double blind, and placebo-controlled	Oral curcumin C3 complex (95% curcuminoids with 70%–80% comprised by curcumin, 15%–25% demethoxycurcumin, and 2.5%–6.5% bisdemethoxycurcumin), capsules taken twice daily with a fatty meal	Placebo-12 2 g/day-12 4 g/day-12	24 weeks	No differences between treatment groups in clinical or biomarker efficacy measures; low native curcumin measured in plasma (7.32 ng/mL)	5/24 subjects withdrew in the curcumin group due to gastrointestinal symptoms	Ringman et al. (2012)
3	60 healthy adults aged 60–85	Randomized, double-blind, and placebo-controlled	400 mg Longvida^®^ optimized curcumin containing approximately 80 mg curcumin in a solid lipid formulation, capsules taken once daily	Placebo-30; Intervention-30	4 weeks	Improved performance on sustained attention and working memory tasks one hour after administration curcumin; better working memory and mood following chronic treatment (4 weeks); reduced total and LDL cholesterol levels	Curcumin treatment was well tolerated and did not significantly impact of any of the examined hematological safety measures	Cox et al. (2015)
4	48 AD patients with moderate dementia	Randomized, double-blind, and placebo-controlled	Unformulated standard curcumin complex with >95% purity (USC); curcumin-galactomannan complex (CGM); 400 mg × 2/day	Placebo-16 USC group-16 CGM group-16	6 months	Improved MMSE and GLFS scores, significant delay in the progress of AD, and improved serum levels of specific biomarkers (BDNF, Aβ42, tau protein, IL-6 and TNF-α) in CGM group	No adverse events were reported in the placebo, USC or CGM groups	Das et al. (2023)
5	60 idiopathic PD patients aged≥30	Randomized, triple-blind, and placebo-controlled	Curcumin nanomicelles in soft gelatin capsules, 80 mg/daily	Placebo-30; Intervention-30	9 months	No significant difference between placebo and curcumin groups	A total of 12 subjects showed nausea and vomiting, gastroesophageal reflux, and dyspepsia in curcumin group while 8 patients were reported with side effects related to gastrointestinal system in placebo group	Ghodsia et al. (2022)
6	96 primiparous women	Randomized, double-blind, and placebo-controlled	500 mg of curcumin capsules, once daily	Placebo-48; Intervention-48	8 weeks	The mean score of depression and anxiety were significantly lower in the intervention group	Four individuals in the intervention group and one person in the placebo group reported gastrointestinal symptoms, such as nausea and stomach pain	Hamlbar et al. (2025)
7	80 patients with diabetic polyneuropathy	Randomized, double-blind, and placebo-controlled	Nano-curcumin capsules (curcumin 72%, desmethoxycurcumin 25%, and bisdemethoxycurcumin 3%), one capsule daily (80 mg)	Placebo-40; Intervention-40	8 weeks	Significant reduction in the mean score of depression and anxiety in the nano-curcumin group; changes in stress score were not statistically significant	Stomachache was reported by two patients	Asadi et al. (2019)
8	42 ALS patients	Double-blind	Brainoil, a dietary supplement containing 600 mg of curcumin, 100 mg of Coenzyme Q10, 300 mg of *Bacopa monnieri*, 250 mg of *Withania somnifera*, 250 mg of *Centella asiatica*, and 1 mg of *Piper nigrum*, once daily	Group A-24: placebo for 3 months (T1), then Brainoil for the following 3 months (T2) Group B-18: only Brainoil for 6 months	6 months	Group B: a stable score of the ALS-FRS-r; reduced AOPPs; stable FRAP exercise values Group A: FRAP exercise values at T1 were reduced and then increased at T2 with introduction of therapy	Gastralgia (N = 3), diarrhea (N = 1), and skin rush (N = 1)	Chico et al. (2018)

AD, Alzheimer’s disease; LDL, low density lipoprotein; MMSE, mini-mental state examination; GLFS, geriatric locomotive function scale; BDNF, brain-derived neurotrophic factor; Aβ, amyloid β; IL, interleukin; TNF, tumor necrosis factor; PD, Parkinson’s disease; ALS, amyotrophic lateral sclerosis; ALS-FRS-r, revised ALS functional rating scale; AOPPs, advanced oxidation protein products; FRAP, ferric reducing antioxidant power.

## 2 Review methodology

The review and research articles were retrieved using relevant keywords (involving curcumin, neurodegenerative diseases, Alzheimer’s disease, Parkinson’s disease, Huntington’s disease, amyotrophic lateral sclerosis, toxic proteins, tau protein, β-Amyloid, alpha synuclein, brain, neuron, apoptosis, metal homeostasis, chelation, ferroptosis, cuproptosis, mitochondria damage, inflammation, oxidative stress, pharmacological effects, neuroprotection, antioxidation/antioxidant, anti-inflammation/anti-inflammatory, mechanism, signal pathway, bioavailability, clinical trial, and so on) from the website and databases including ScienceDirect, Google Scholar, Web of Science, ResearchGate, and PubMed. For the included clinical studies of curcumin in the treatment of neurological diseases, the necessary information was required to be reported, including the clinical trial registration, the inclusion criteria of subjects, the basic information of drugs (source, composition, formulation type, dosage and administration method), placebo, treatment time, measured indicators, outcomes, side effects, etc. More than 200 articles published mainly from 2015 to 2025 were selected in this way. Then the useful information related to the topic of this paper was extracted and summarized from these collected references.

## 3 Pharmacological effects of curcumin

### 3.1 Antioxidation

Oxidative stress is a critical factor contributing to the onset and progression of NDs. It arises from an imbalance between the production of reactive oxygen species (ROS) and the body’s antioxidant defenses (Keeney et al., 2024). ROS, such as superoxide anions, hydrogen peroxide and hydroxyl radicals, are byproducts of normal cellular metabolism, particularly mitochondrial respiration. Under physiological conditions, ROS levels are tightly regulated by endogenous antioxidant systems, including superoxide dismutase (SOD), catalase (CAT) and glutathione peroxidase (GPx) (Li et al., 2025). However, excessive ROS and impaired antioxidant mechanisms will cause oxidative damage to lipids, proteins, DNA and other cellular components (Dorszewska et al., 2023). In neurons, oxidative stress disrupts mitochondrial function, leading to reduced ATP (adenosine triphosphate) and increased ROS, which forms a vicious cycle of oxidative damage. Cell homeostasis is thus destroyed, and the misfolding and aggregation of toxic proteins are exacerbated, like Aβ in AD and α-syn in PD (Van Laar et al., 2020; Meng et al., 2011). Oxidative stress induces lipid peroxidation, and impairs the structural and functional integrity of cell membranes and organelles. Moreover, the damage to DNA caused by excessive ROS activates apoptotic pathways to accelerate neuronal cell death (Al Olayan et al., 2020). The imbalance between ROS and cellular defense aggravates neuroinflammation by stimulating microglia and astrocytes to release pro-inflammatory cytokines (Wang et al., 2021a; Dash et al., 2025). The blood-brain barrier is also impaired under such conditions, losing the ability to prevent peripheral immune cells and harmful molecules from infiltrating the central nervous system (CNS), which thereby accelerates neurodegeneration (Yang et al., 2024; Kim S. et al., 2024). The cumulative impact of these processes results in the gradual loss of neuronal structure and function, manifesting as cognitive decline, motor impairments and other degenerative symptoms associated with NDs.

Curcumin exerts the antioxidant effects through multiple pathways. Firstly, it can directly scavenge ROS and reactive nitrogen species, thereby reducing oxidative damage to cellular components (Alisi et al., 2018). It is attributed to the well-characterized structural motifs (Ahmed et al., 2024). The chemical structure including two phenolic rings connected by a β-diketone moiety enables curcumin to neutralize free radicals and inhibit lipid peroxidation. The activity of endogenous antioxidant enzymes such as SOD, CAT and GPx is enhanced by curcumin, which plays a crucial role in detoxifying ROS and maintaining cellular redox homeostasis (Santana-Martínez et al., 2019). In addition to the direct antioxidant effects, curcumin can also regulate the signaling pathways of antioxidant reactions to protect neurons from damage (Duan et al., 2022). For instance, nuclear factor erythroid 2-related factor 2 (Nrf2) is a master regulator of antioxidant responses. Under normal conditions, Nrf2 is sequestered in the cytoplasm by the inhibitor, Kelch-like ECH-associated protein 1 (Keap1). When subjected to oxidative stress, Nrf2 translocates to the nucleus and binds to antioxidant response elements (ARE), inducing the expression of antioxidant genes (Kang et al., 2024). Curcumin can promote the dissociation of Nrf2 from Keap1 and enhance its activation to facilitate the upregulation of antioxidant enzymes and phase II detoxifying enzymes, which collectively reduce oxidative stress and strengthen cellular resilience (Rathore et al., 2023). On the other hand, nuclear factor-kappa B (NF-κB) is a transcription factor that promotes the expression of pro-inflammatory cytokines and related enzymes such as cyclooxygenase, inducible nitric oxide synthase and nicotinamide adenine dinucleotide phosphate (NADPH) oxidase (Patel et al., 2017). Cysteine-alanine-glutamine-lysine peptide-modified antioxidant nanoparticles were developed for co-delivery of curcumin (C-PPS/C) to modulate oxidative and neuroinflammatory disturbances of mice with traumatic brain injury. It was revealed that C-PPS/C nanoparticles accumulated at the injury site and broke the “ROS-neuroinflammation” cycle to protect the blood-brain barrier and promote long-term neurological recovery. The hydrophobic core of C-PPS/C composed of poly (propylene sulfide) can react with and scavenge ROS, while curcumin release further suppressed ROS and inflammation. Meanwhile, C-PPS/C nanoparticles were found to inhibit the NF-κB pathway to reduce the expression of pro-inflammatory genes (Fu X. et al., 2025). Additionally, curcumin’s ability to chelate metal ions like iron and copper further contributes to the antioxidant effects, as these metals are known to catalyze the production of highly reactive hydroxyl radicals through Fenton reactions (Cengiz et al., 2023). For example, Cd exposure (2.5 mg/kg for 60 days) induced behavioral impairments in mice through prefrontal cortex cellular inflammation and oxidative stress, as a result of the reduction of endogenous antioxidant enzyme activity, and the increase of pro-inflammatory markers (IL-6 and TNFα) and decrease of anti-inflammatory cytokine IL-10. Curcumin treatment (20–160 mg/kg for 30 days) could mitigate these behavioral and biochemical impairments by reducing the neuroinflammation and oxidative damage as well as increasing the number of viable prefrontal cortex neuronal cells (Namgyal et al., 2021).

### 3.2 Anti-inflammation

Inflammation is triggered by body’s defense responses to stimuli such as injury and infection, and is usually conducive to tissue repair and pathogen clearance. Acute inflammation is always short-term and beneficial, but the chronic inflammation, caused by persistent triggering or immune dysregulation, can damage tissues and lead to neurodegeneration (Zhang W. et al., 2023). Neuroinflammation is characterized by the activation of microglia and astrocytes, and the release of pro-inflammatory cytokines (Gulen et al., 2023). Microglia are resident immune cells in CNS, which perform the functions of synaptic pruning, damage repair, homeostasis maintenance and cell phagocytosis. However, facing pathological stimuli such as protein aggregates, microglia are activated and adopt a pro-inflammatory phenotype, leading to the release of cytokines involving interleukin-1β (IL-1β), interleukin-6 (IL-6) and tumor necrosis factor-alpha (TNF-α) to exacerbate neuronal impairment (Peruzzotti-Jametti et al., 2024; Nikolopoulos et al., 2023). The activation of microglia is regulated by pattern recognition receptors (PRRs), including Toll-like receptors (TLRs) and nucleotide-binding and oligomerization domain (NOD)-like receptors (NLRs), which can recognize pathogen-associated molecular patterns (PAMPs) and damage-associated molecular patterns (DAMPs) (Li and Wu, 2021). Astrocytes are another type of glial cells related to neuroinflammation. In response to inflammatory signals, astrocytes undergo reactive astrogliosis, accompanied by cellular hypertrophy and the upregulation of glial fibrillary acidic protein (GFAP) (Lopez Rodriguez et al., 2021; Linard et al., 2023). Reactive astrocytes release cytokines and chemokines that further amplify the inflammatory response and disrupt the blood-brain barrier, allowing peripheral immune cells to infiltrate the CNS (Ghita et al., 2021; Qian et al., 2023). The chronic activation of microglia and astrocytes leads to a self-perpetuating cycle of inflammation and neurodegeneration. Pro-inflammatory cytokines and ROS not only damage neurons directly but also induce the misfolding and aggregation of toxic proteins, a hallmark event of NDs. Additionally, inflammation destroys synaptic plasticity and inhibits the clearance of harmful substances to further aggravate neuronal dysfunction (Geng et al., 2020; Zhang et al., 2014). In summary, the activation of microglia and astrocytes, and the subsequent release of pro-inflammatory cytokines and ROS, create an unfavorable environment for neuronal damage and death.

Regulating inflammatory signaling pathways and inhibiting inflammatory mediators are the main mechanisms of curcumin to achieve anti-inflammatory effects. By inhibiting the activity of the inhibitory kappa B kinase (IKK), curcumin prevents the translocation and activation of NF-κB, thereby reducing the expression of chemokines and pro-inflammatory cytokines such as TNF-α, IL-1β and IL-6, which ultimately attenuates inflammatory responses (Jobin et al., 1999). NOD-like receptor protein 3 (NLRP3) inflammasome is a protein complex involved in the development of immune and inflammation-related diseases. Curcumin can directly restrain the assembly of NLRP3 inflammasome, or inhibit its activation by restricting the NF-κB pathway (Cai et al., 2025; Ruan et al., 2022). T helper 17 cells (Th17), one of the targets of curcumin, are an important pro-inflammatory cell that secretes IL-17, IL-22 and IL-23 to promote inflammation responses. On the contrary, regulatory T cells (Tregs) possess prominent anti-inflammatory effects. The imbalance of Th17 and Tregs can lead to an abnormal immune response (Chaudhry et al., 2011). Curcumin suppresses Th17 differentiation and regulates Treg/Th17 rebalance by inhibiting IL-23/Th17 pathway to maintain immune homeostasis. The expression of Th17 cells related cytokine profiles (IL-17A and RORγt) was dramatically decreased in curcumin-treated mice, indicating the inhibited differentiation and development of Th17 cells. Besides, it was demonstrated that type 4 metabotropic glutamate receptor (mGluR4) was constitutively expressed in mouse bone marrow-derived dendritic cells (BMDC), and mGluR4 siRNA-transfected BMDC tipped the balance of T cell differentiation toward the Th17 phenotype. Curcumin upregulated mGluR4 expression in mouse BMDC, which likely contributed to the mechanism of inhibiting the Th17 cell differentiation (Zhao et al., 2017; Rahimi et al., 2019). Mitogen-activated protein kinases (MAPKs) are an pivotal group of regulatory factors in cellular stress response. Inhibiting the MAPK pathway is another way for curcumin to reduce the release of inflammatory mediators and cell apoptosis (Maugeri et al., 2023). It was recently found that curcumin interacted with the Z1-type protein tyrosine phosphatase receptor (PTPRZ1) to maintain the enzymatic activity, thereby regulating the phosphorylation of the m6A-reader YTH domain-containing family protein 2 (YTHDF2) and avoiding the overactivation of microglia (Zhang et al., 2025). This process is beneficial to relieve neuroinflammation, alleviate brain damage and promote neuronal repair. In addition to the above strategies, curcumin inhibits inflammation-related transcription factors by reducing the accumulation of ROS (Chen T. et al., 2024). Autophagy-related pathways (such as AMPK/mTOR) can be also activated to promote the clearance of toxic protein aggregates and damaged cells, so as to avoid inflammation and neuronal damage (Khayatan et al., 2025; Fu Y. et al., 2025).

### 3.3 Apoptosis inhibition

Apoptosis, also called programmed cell death, is a highly regulated process that automatically eliminates damaged or unnecessary cells to maintain tissue homeostasis (Baar et al., 2017). Apoptotic cells are characterized by distinct morphological changes, including cell shrinkage, chromatin condensation, DNA fragmentation, and the formation of apoptotic bodies which are subsequently phagocytosed by neighboring cells (Arvanitaki et al., 2024). However, when apoptosis is dysregulated as a result of oxidative stress, neurotrophic factor deficiency, inflammatory responses, and protein misfolding and aggregation, it can induce excessive and premature cell death, thereby contributing to progressive neuronal loss (Kumari et al., 2023; Norton et al., 2024). The principal factors of apoptosis program include Apaf-1 (apoptotic protease-activating factor 1), Bcl-2 (B cell lymphoma-2) family proteins and caspases (cysteinyl aspartate-specific proteinases) (Qin et al., 1999). The Bcl-2 family, comprising both anti-apoptotic proteins (like Bcl-2 and Bcl-xl) and pro-apoptotic proteins (like Bax and Bak), governs apoptosis primarily by modulating mitochondrial permeability. Under normal physiological conditions, anti-apoptotic proteins, such as Bcl-2 and Bcl-xl, form homodimers or multimers on the mitochondrial outer membrane to maintain its integrity and inhibit cytochrome c release. In contrast, pro-apoptotic Bax and Bak predominantly exist as inactive monomers (Edlich et al., 2011). Upon exposure to apoptotic stimuli, Bax and Bak undergo conformational changes, become activated, and insert into the mitochondrial membrane, which then heightens mitochondrial permeability and facilitates the release of cytochrome c (Antonsson et al., 1997). The released cytochrome c subsequently binds to Apaf-1 to generate the apoptosome that recruits and activates caspase-9 as well as downstream effector caspases (like caspase-3/caspase-6), ultimately executing cell death (Kim et al., 2005; Li et al., 1997). Bcl-2 can interact with Bax to form heterodimers, thereby suppressing Bax activity and cytochrome c release to block the subsequent apoptotic cascade. Excessive accumulation of toxic proteins induces endoplasmic reticulum stress to disrupt calcium homeostasis, activating the apoptotic pathway (Lim et al., 2023). Additionally, neurofibrillary tangles formed by hyperphosphorylated tau proteins reduce their binding affinity with neuronal tubulin, destabilizing microtubules and promoting neurodegeneration (Savastano et al., 2021; McMillan et al., 2023). By secreting Il-1α, TNF and complement component subunit 1q, the activated microglia induce the formation of neurotoxic reactive astrocytes which lose the ability to support neuronal survival, outgrowth, synaptogenesis and phagocytosis, leading to the death of neurons and oligodendrocytes (Zhang L. et al., 2023). Furthermore, reduced levels of neurotrophic factors, such as brain-derived neurotrophic factor (BDNF), diminish the initiation of anti-apoptotic process, rendering neurons more vulnerable to apoptosis (Zhang K. et al., 2023).

Curcumin, on the one hand, scavenges ROS to improve neuronal microenvironment and prevent apoptosis; on the other hand, it promotes microglial polarization toward an M2 phenotype and inhibits M1 polarization, thereby suppressing inflammation-induced apoptosis and fostering neuronal regeneration (Qian et al., 2024; Chen Z. et al., 2024). The expression of pro-apoptotic proteins Caspase 3/8/9, Bax and apoptotic peptidase activating factor 1 is reduced by curcumin while that of anti-apoptotic proteins Bcl-2 is increased. Endoplasmic reticulum stress is mitigated by curcumin-mediated downregulation of Grp78 (glucose-regulated protein 78) and CHOP (C/EBP homologous protein) expression. This effect is likely attributed to curcumin’s ability to upregulate CAT and glutathione reductase levels, thereby enhancing cellular antioxidant capacity. The resulting reduction in ROS overproduction helps preserve the functional integrity of mitochondrial respiratory chain complexes I and II (Wu et al., 2024). The combination of curcumin and vagus nerve stimulation was found to significantly lower the neurological deficits, infract volume, neural apoptosis and inflammatory cytokines release. The phosphorylation levels of AKT and ERK2 were both increased after the combination therapy (Xu et al., 2018). Mitochondrial damage and dysfunction are key factors of neuron loss. Curcumin protects mitochondrial respiratory chain by maintaining the stability of membrane potential. At the same time, mitochondrial dysfunction is ameliorated via promoting mitochondrial biogenesis to inhibit abnormal neuronal apoptosis (Zheng et al., 2023). Excessive autophagy can lead to metabolic stress and degradation of cellular components, which exacerbates the structural destruction and functional decline of tissues (Aman et al., 2021). It was indicated that curcumin significantly reduced the level of H_2_O_2_-induced autophagy with a decreased expression of autophagy marker proteins LC3-II and Beclin-1. The ERK1/2 signaling pathway was also inactivated by inhibiting phosphorylation, thereby reducing autophagy responses and protecting nerve cells from oxidative stress (Ruan et al., 2024). Curcumin (200 mg/kg) significantly ameliorated brain injury and neurological deficits in rats subjected to middle cerebral artery occlusion by upregulating autophagy-related proteins p-Akt and p-mTOR, while downregulating autophagy-associated proteins LC3-II/LC3-I and inflammation-related proteins IL-1, TLR4, p-38 and p-p38. However, these protective effects were attenuated when either LY294002 (a specific inhibitor of the PI3K/Akt/mTOR pathway) or anisomycin (an activator of the TLR4/p38/MAPK pathway) was administered. It was suggested that curcumin exhibited neuroprotective effects by modulating autophagic activities via the PI3K/Akt/mTOR signaling pathway and simultaneously suppressing inflammatory responses through regulation of the TLR4/p38/MAPK pathway (Huang et al., 2018). In summary, the excessive loss of neurons is avoided by curcumin through multiple mechanisms to prevent progressive neurodegeneration.

### 3.4 Metal chelation

Metal ions are widely present in biological systems and participate in many key biochemical processes including material transport, energy conversion, information transmission and metabolic regulation (Chen et al., 2022; Maniscalchi et al., 2024). Common ions, like zinc (Zn^2+^), magnesium (Mg^2+^), iron (Fe^2+^/Fe^3+^) and copper (Cu^2+^), are co-factors of a variety of enzymes which play an important role in stabilizing enzyme conformation, transferring electrons, and connecting enzymes and substrates in enzymatic reactions (Valdez et al., 2014). They are indispensable in human bodies. However, the imbalance of metal ions, whether deficiency or excess, can lead to metabolic disorders, cell and tissue damage, weakened immunity and neurological dysfunction (Tian et al., 2024). The accumulation of metal ions promotes the production of hydrogen peroxide and hydroxyl radical through Fenton and Haber-Weiss reactions, resulting in lipid peroxidation of cell membrane (von Krusenstiern et al., 2023). Cellular integrity is damaged by the oxidative damage of lipids, proteins and DNA (Khan et al., 2020). Metal ion imbalance and oxidative stress trigger the overproduction of Aβ by activating β- or γ-secretases and inhibiting α-secretase, and also cause tau hyperphosphorylation by activating protein kinases, such as glycogen synthase kinase-3β (GSK-3β), cyclin-dependent protein kinase-5 and MAPKs, and inhibiting protein phosphatase 2A (Wang et al., 2020). It has been found that both essential (Mn, Fe, Cu and Zn) and nonessential (Pb, Al and Cd) metals can accumulate in the brain and activate microglia and astrocytes to release pro-inflammatory factors (Domingo-Relloso et al., 2024). The inflammation and oxidative stress intensify each other, forming a vicious cycle. In addition, metal ion imbalance interferes with the electron transport chain in mitochondria, leading to the loss of membrane potential and energy metabolism disorders. The corresponding decrease in ATP production eventually leads to cell apoptosis (Shen et al., 2023). Calcium ions (Ca^2+^) are indispensable to physiological activities, which participate in the signal transduction, neurotransmitter release and hormone secretion. Excessive metal ions impair Ca^2+^ channels and calmodulin function, generating abnormally elevated intracellular calcium concentrations. The breakdown of calcium homeostasis affects the synthesis, release and reuptake of neurotransmitters, followed by excitotoxicity and loss of neurons (Fan et al., 2020; Bharat et al., 2023).

Ferroptosis and cuproptosis are two specific modes of cell death that depend on the exposure to iron and copper, respectively (Zou et al., 2020; Li S. et al., 2022). The ferroptosis is characterized by iron metabolism disorder, ROS accumulation, reduced glutathione levels and the inactivation of glutathione peroxidase 4 (GPX4). The presence of Fe^2+^ and lipoxygenases facilitates the lipid peroxidation of cell membranes and ultimately induces ferroptosis (Qiu et al., 2024). Both exogenous and endogenous pathways drive this process. Exogenous pathways rely on the inhibition of system Xc– (cystine/glutamate transporter) and the activation of the transferrin and transferrin receptor 1, while the endogenous pathway mainly involves blocking the activation of GPX4 (Xue et al., 2023). As for cuproptosis, it is closely related to mitochondrial metabolism. The excess copper binds to lipase in the mitochondrial TCA cycle, which accelerates protein aggregation and proteotoxic stress, leading to cell death (Chen et al., 2022). It has been proved that changes of iron and copper homeostasis is directly linked to the progression of multiple NDs, including AD, HD and ALS (Maniscalchi et al., 2024).

The metal chelation of curcumin is mainly attributed to the unique chemical structure, including two adjacent phenolic hydroxyl groups and a β-diketone moiety, which enable it to form stable complexes with various metal ions through coordination covalent bonds (Mari et al., 2022). The oxygen atoms in phenolic hydroxyl groups provide lone pair electrons to interact with positively charged metal ions, and the carbonyl oxygen atoms in the β-diketone structure further enhance the stability of metal-curcumin complexes by providing additional coordination sites for metal ions (Moradi et al., 2020). The metal-chelating ability is affected by many factors, especially the pH. Under acidic conditions, the phenol hydroxyl groups are more readily protonated, thereby increasing the affinity to metal ions (Wahyudi et al., 2022; Rasouli and Ghavami, 2021). Neuroprotective effects are exerted by modulating the activity of metal-dependent enzymes, reducing the production of ROS, and inhibiting the aggregation of abnormal proteins. The chelating effect of curcumin reduces the levels of free metal ions and inhibits their participation in the reactions of free radical generation, which helps protect neurons from oxidative stress-induced cell death (Namgyal et al., 2021). Additionally, the aggregation of toxic proteins is also reduced, slowing the progression of neurodegeneration. The binding of cadmium or arsenic to SOD diminishes its enzymatic activity, whereas curcumin forms hydrogen bonds with histidine residues, thereby reducing SOD’s affinity for arsenic and cadmium and restoring its activity (Cengiz et al., 2023). Cerebral cortex acetylcholinesterase (AChE) and adenosine deaminase (ADA) activities were elevated in rats exposed to Cd (2.5 mg/kg). Co-treatment with curcumin reversed these enzyme activities back to control levels. Furthermore, Cd exposure induced an increase in lipid peroxidation in the cerebral cortex, accompanied by a reduction in functional sulfhydryl (-SH) groups and nitric oxide (NO), a critical neurotransmitter and neuromodulatory agent. Co-administration of curcumin (12.5 or 25 mg/kg) enhanced the non-enzymatic antioxidant status and NO levels while simultaneously reducing malondialdehyde levels. Therefore, the inhibition of AChE and ADA activities, along with the enhancement of the antioxidant status mediated by curcumin, may provide potential mechanisms underlying its cognitive-enhancing effects in Cd-induced memory dysfunction (Akinyemi et al., 2017). Curcumin can also improve the cell apoptosis caused by metal toxicity. Excessive accumulation of heavy metals promoted the cleavage of poly (ADP-ribose) polymerase-1 and caspase-3, suppressed Bcl-2 expression, and induced the release of cytochrome c and second mitochondria-derived activator of caspases from the mitochondria into the cytosol. Curcumin inhibited both early and late apoptosis induced by heavy metals and reversed the alterations in apoptotic markers. Metallothionein (MT), a cysteine-rich protein, plays a pivotal role in metal detoxification. Curcumin increased the level of MT2A mRNA and enhanced the expression of MT2. The antiapoptotic effects of curcumin were attenuated under MT2A knockdown conditions, suggesting that MT2A may be a critical target of curcumin (Wahyudi et al., 2022). Aluminum is known as a neurotoxic metal, which may show negative effects on the nervous system, particularly at higher concentrations (Bishop et al., 1997). The symptoms include movement disorders, seizures, memory loss, learning disabilities and depressive tendencies. Curcumin has the potential of reducing the bonding affinity of Al^3+^ to DNA and preventing the Al^3+^-induced cognitive impairment (Sood et al., 2012). It can be concluded the metal-chelating ability of curcumin, driven by its unique chemical structure, plays a crucial role in neuroprotection by modulating enzyme activity, reducing oxidative stress and inflammation, inhibiting toxic protein aggregation, and alleviating metal-induced apoptosis, thereby offering a promising therapeutic strategy for NDs.

## 4 Function of curcumin in the treatment of specific NDs

### 4.1 Alzheimer’s disease

#### 4.1.1 Pathogenesis of AD

Alzheimer’s disease (AD) is a progressive neurodegenerative disorder that accounts for 60%–80% of all dementia cases worldwide. The main manifestations are behavioral changes and gradual decline of cognitive functions, which seriously affect daily life (Self and Holtzman, 2023). It is estimated that over 50 million people are currently living with AD, and this number is projected to rise to 152 million by 2050 (Long and Holtzman, 2019). The prevalence of AD increases with age. Approximately 5%–10% of individuals over 65 years old and more than 20% of those over 85 years old are affected. With the aging of global population, AD has become a major public health challenge (Pichet Binette et al., 2020). Hallmark pathological features of AD are the accumulation of Aβ plaques and neurofibrillary tangles composed of hyperphosphorylated tau proteins (Islam et al., 2025). Aβ plaques are formed due to the abnormal cleavage of amyloid precursor protein by β- and γ-secretases that leads to the production and aggregation of toxic peptides in brain. The plaques disrupt neuronal communication and trigger inflammatory responses (Pang et al., 2022). Tau, on the other hand, is a structural protein mainly distributed in CNS, which is responsible for maintaining the stability of microtubules, like the “skeleton of neurons”. It facilitates the efficient transport of nutrients to support the optimal exercise of physiological functions (Roemer-Cassiano et al., 2025). However, in AD, tau becomes hyperphosphorylated to detach from microtubules and form toxic neurofibrillary tangles that impair cellular transport and accelerate neuronal death (Berlind et al., 2025). The upregulation of histone deacetylase 7 (HDAC7) induces AD-like tau pathologies by deacetylating the transcriptional factor EB (TFEB) and inhibiting lysosomal biogenesis in astrocytes. Suppressing HDAC7-TFEB signaling is promising for alleviating AD and other neurodegenerative disorders (Ye et al., 2025). The recent findings also suggested that Aβ promoted tau spreading in AD by eliciting neuronal hyperactivity and hyperconnectivity (Roemer-Cassiano et al., 2025). These pathological changes primarily affect the hippocampus and cerebral cortex, which are critical regions for memory and cognitive functions.

Oxidative stress, neuroinflammation driven by activated microglia and astrocytes, mitochondrial dysfunction and genetic variation are all key factors in the development of AD (Long and Holtzman, 2019). It has been found that mutations in the amyloid protein precursor (*APP*), presenilin-1 (*PSEN1*), and presenilin-2 (*PSEN2*) genes are associated with autosomal dominant early-onset familial AD, while apolipoprotein E4 (*APOE4*) represents the most significant genetic risk factor for late-onset sporadic AD, contributing to the deposition of Aβ peptides and hyperphosphorylated tau proteins (Giau et al., 2019; Rao et al., 2025). Accumulating evidence has unveiled the bidirectional communication between gut microbiome and CNS, referred to as the “microbiota-gut-brain axis” (Forner et al., 2021; Wang X. et al., 2019; Boehme et al., 2021). The gut microbiome regulates microglial maturation and activation via short-chain fatty acids (SCFAs) release. The depletion of SCFAs stimulates the transition of cells towards a pro-inflammatory state. The elevated immune activation is coupled with impaired hippocampal synaptic transmission, and cholinergic gamma oscillations (Loh et al., 2024). *Akkermansia muciniphila*, a mucin- and SCFA-yielding intestinal microorganism, is essential for the regulation of metabolic homeostasis and immune system, and propionic acid is one of the main metabolites. It was revealed that propionate reduced mitochondrial over-division by downregulating mitochondrial fission protein (DRP1) via G-protein coupled receptor 41, and activated PINK1/PARKIN-mediated mitophagy via GPR43 to maintain mitochondrial homeostasis and improve the pathological injury of AD (Wang Z. et al., 2025).

#### 4.1.2 Function of curcumin in AD

The strategies of curcumin to address AD mainly include the inhibition of Aβ aggregation and tau hyperphosphorylation, anti-oxidation, anti-inflammation and brain-gut axis regulation (Pei et al., 2024; Ruan et al., 2024). Curcumin directly binds to small Aβ peptides to block the aggregation and fibril formation, which attenuates their destructive effect on cell membranes, thereby reducing cytotoxicity and improving cell survival (Chen Z. et al., 2024; Zhang et al., 2010). Moreover, curcumin facilitates the clearance of Aβ by enhancing microglial phagocytic activity and up-regulating Aβ-degrading enzymes. Neprilysin (NEP) is the most important Aβ-degrading enzyme. Its expression decreases with age and is negatively correlated with Aβ accumulation (Iwata et al., 2001; Kim et al., 2023). The investigation of 25 curcumin analogs surprisingly showed that dihydroxylated curcumin, monohydroxylated demethoxycurcumin, and mono- and di-hydroxylated bisdemethoxycurcumin, increased NEP activity, but curcumin did not. Thus, it was proposed that polyhydroxycurcuminoids might be better candidates than curcumin as a dietary supplement in the prevention and therapy of AD (Chen et al., 2016). Exposing organotypic hippocampal cultures to Aβ, curcumin and/or LY294002, it was found that curcumin treatment mitigated the Aβ-induced decline in synaptophysin expression, and significantly reduced cell death. Aβ resulted in the increase of phosphorylated (Ser45) β-catenin and the decrease of β-catenin immunocontent, but curcumin prevented this destabilization. Additionally, the neuroprotective effects of curcumin was inhibited by LY294002, and the phosphorylation/activation of Akt and the phosphorylation/inactivation of GSK-3β were activated, which revealed that the neuroprotection may involve the PI3-K pathway and β-catenin signaling, a key transduction factor of Wnt signaling pathway (Hoppe et al., 2011). Metabolomic studies of other natural compounds, have similarly identified hippocampal and cortical metabolic pathways critical for counteracting Aβ-induced neurotoxicity, reinforcing the potential of phytochemicals in multi-target AD therapies (Li H. et al, 2022). On the other hand, curcumin inhibits the activation of NLRP3 inflammasomes in neurons and microglia to relieve neuroinflammation and reduce the amyloid plaque burden in AD (Guglielmo et al., 2017; Ruan et al., 2022). Molecular docking indicated that there were putative binding sites with curcumin in microtubule-binding regions of tau. It could not only inhibit the oligomerization of tau, but also disintegrate preformed tau filaments (Rane et al., 2017). The curcumin-primed exosomes efficiently penetrate blood-brain barrier through receptor-mediated transcytosis to access brain tissues, and inhibit tau phosphorylation by activating the AKT/GSK-3β pathway, which holds great potential in improving targeted drug delivery and restoring neuronal function (Wang H. et al., 2019). In the initial stage of AD, copper accumulation triggers a self-assembly of both Aβ40 and Aβ42 (Tian et al., 2024). Under inflammatory conditions, high levels of copper in serum can also induce oxidative stress and promote inflammatory response. The difluoroboron curcumin derivative (DF-Cur) showed highly specific and selective recognition of Cu^2+^, which could serve as a diagnostic agent for the copper detection. Additionally, DF-Cur promoted the excretion of Cu^2+^ and reduced its accumulation *in vivo*, alleviating the damage to nervous system (Wang M. et al., 2021). It was demonstrated that curcumin functioned as prebiotics, which adjusted the gut microbiota community, improved intestinal barrier integrity, and prevented the release of detrimental metabolites and pro-inflammatory factors. Considering the link between gut health and neurodegeneration, the treatments targeting gut microbiota may be a promising strategy to address neuroinflammation and delay AD (Kim Y. et al., 2024; Su J. et al., 2021).

Although curcumin has demonstrated promising anti-AD activity in cellular and murine models, consistent clinical evidence remains lacking. In a clinical trial involving 36 patients with mild-to-moderate AD, no significant differences were observed between oral curcumin (2 or 4 g/day) and placebo after 24 weeks of treatment (Ringman et al., 2012). Pretty low native curcumin (7.32 ng/mL) was measured in the plasma. Gastrointestinal adverse effects were reported in five participants from the intervention group. Conversely, a separate six-month randomized controlled trial revealed that a curcumin-galactomannan complex (400 mg twice daily) significantly improved Mini-Mental State Examination (MMSE) and Geriatric Locomotive Function Scale (GLFS) scores of AD patients compared to both placebo and unformulated standard curcumin, suggesting a potential delay in disease progression (Das et al., 2023). This formulation positively modulated serum levels of AD-specific biomarkers, including BDNF, Aβ42, tau protein, IL-6 and TNF-α. Notably, no adverse effects were reported in either the curcumin or placebo groups in this trial. These findings highlight that the clinical efficacy of curcumin may depend on formulation type, dosage and bioavailability. Further large-scale, long-term studies are warranted to conclusively establish its therapeutic effects and safety profile in AD populations.

### 4.2 Parkinson’s disease

#### 4.2.1 Pathogenesis of PD

Parkinson’s disease (PD) is the second most prevalent neurodegenerative disorder after AD, mainly manifested as motor dysfunction and cognitive decline. Globally, more than 10 million individuals currently suffer from PD, with a prevalence rate of 1%–2% among those over 65 years old (Bloem et al., 2021; Ben-Shlomo et al., 2024). PD can be categorized into two distinct subtypes, familial (genetic) PD and sporadic PD, based on the etiological origins. Familial PD results from well-defined pathogenic mutations in associated genes (like *SNCA*, *LRRK2*, *PRKN*, *PINK1* and *DJ-1*). These mutations exhibit autosomal dominant or recessive inheritance patterns and can be transmitted across generations, often leading to multiple affected individuals within families. As for sporadic PD which accounts for approximately 90% of cases, it lacks clear familial aggregation. The pathogenesis involves complex interactions between environmental exposure, lifestyle and age. The primary pathogenesis of PD involves the degeneration and death of dopaminergic neurons in midbrain substantia nigra, as well as the presence of abundant filamentous inclusions of α-syn in the form of Lewy bodies and Lewy neurites (Yang et al., 2022; Mahul-Mellier et al., 2020). As a widely expressed protein in CNS, α-syn normally plays a role in neuronal signal transduction and synaptic function. However, in PD, α-syn monomers undergo misfolding and aggregate into fibrous structures that transfer between neurons and rapidly spread throughout brain regions, causing functional damage and neuronal death (Hallacli et al., 2022). Toxic proteins infiltrating the midbrain substantia nigra can trigger the demise of dopaminergic neurons, leading to motor symptoms such as bradykinesia, resting tremor, and muscle rigidity. When they disseminate to the cerebral cortex, cognitive deficits like memory decline, anxiety and somnipathy may emerge (Park et al., 2025).

*FAM171A2* is a key regulatory gene of progranulin, a secreted pleiotropic glycoprotein associated with NDs (Xu W. et al., 2020). The expression of *FAM171A2* was found to be significantly elevated in PD and positively correlated with the aggregation of α-syn. The domain 1 of FAM171A2 protein is tightly bound to the C-terminal region of α-syn by electrostatic interaction, and the binding degree of FAM171A2 to pathological α-syn fibers is much stronger than that of monomeric α-syn. Their combination enhances the endocytosis of neurons, encouraging toxic proteins to enter the cells (Wu et al., 2025). The endosomal sorting complex required for transport (ESCRT) system is responsible for the sorting, trafficking and degradation of proteins and other cellular components. It is critical in facilitating the degradation of ubiquitinated α-syn through the endosome pathway (Nim et al., 2023). The E3 ubiquitin ligase Listerin promotes K27-linked polyubiquitination of α-syn, directing it to the endosome for the subsequent degradation. It has been revealed that the deletion of the Listerin gene exacerbates α-syn-induced olfactory dysfunction and motor deficits in a mouse model of PD (Qin et al., 2025). Parthanatos-associated apoptosis-inducing factor nuclease (PAAN), also known as macrophage migration inhibitory factor (MIF), is a critical member of the PD-D/E(X)K nuclease family that serves as a terminal executor in the parthanatos pathway (Wang et al., 2016). Neurodegeneration is triggered by pathological α-syn through the nuclease activity of PAAN/MIF. Both genetic ablation of PAAN/MIF and the nuclease-deficient mutant have been demonstrated to protect against dopaminergic neuron loss and mitigate behavioral impairments in the α-syn preformed fibril mouse model of sporadic PD (Park et al., 2022).

Epigenetic mechanisms, including DNA methylation, histone modifications and non-coding RNA regulation, play significant roles in modulating dopamine transporter (DAT) endocytosis, a process implicated in PD pathogenesis (Liang et al., 2025). While no direct epigenetic regulator of DAT endocytosis has been identified, DNA methyltransferases can indirectly affect this process by altering the expression of genes involved in key signaling pathways, such as protein kinase C and D2 receptor systems (Cremona et al., 2011; Su and Liu, 2017). Similarly, RNA-modifying enzymes, particularly m6A methyltransferases, influence DAT endocytosis by regulating the synthesis and functionality of associated proteins (Jiang et al., 2021). Histone-modifying enzymes contribute to this regulatory network by controlling chromatin accessibility, thereby affecting the expression of genes critical for endocytosis, endosome formation and recycling process (Wang H. et al., 2021). Collectively, these epigenetic modifications establish a sophisticated regulatory network that indirectly controls the dynamics of DAT endocytosis and recycling, representing a crucial regulatory node in neurotransmitter transport and PD progression.

Genetic variation, including single nucleotide variants, small indels, structural variants, copy number variants and short tandem repeats, is another important cause of PD, which is associated with about 10% of cases (Wang C. et al., 2024; Hop et al., 2024). For example, the variant of *LRRK2* is a risk factor for PD in Chinese and Korean populations, corresponding to *SNCA* and *MAPT* genes in Europeans and *GBA1* in Africans (Rizig et al., 2023). *SNCA* is responsible for encoding α-syn. Its mutation can cause the overexpression and aggregation of α-syn, resulting in the formation of Lewy bodies. The leucine-rich repeat kinase 2 (LRRK2) is a multifunctional protein that coordinates a variety of cellular processes, including vesicle transport, autophagy, lysosomal degradation, neurotransmission and mitochondrial activity (Zhu et al., 2023; Deniston et al., 2020). Gain-of-function mutations of *LRRK2* gene trigger lysosomal dysfunction and abnormal cellular response to membrane damage, leading to vesicular transport dysfunction, neuroinflammation, accumulation of alpha-synuclein, mitochondrial dysfunction and ciliary loss, ultimately leading to PD (Gustavsson et al., 2024; Simuni et al., 2020). In addition to the above factors, the identified mechanisms of PD also include oxidative stress, ferroptosis, gut dysbiosis and so on. Their interaction complicates the disease and poses great challenges to clinical treatment and drug development (Dong-Chen et al., 2023; Kotsiliti, 2023).

#### 4.2.2 Function of curcumin in PD

Up to now, the research on curcumin in the treatment of PD has been mainly conducted at the molecular level and in mouse models. There is still a lack of large-scale human experiments and convincing clinical evidence. Curcumin mitigates neuronal degeneration by blocking the histone deacetylase 6 (HDAC6)-NLRP3 signaling pathway to suppress NLRP3 inflammasome-mediated inflammatory responses and counteracts α-syn-induced neurotoxicity (Cai et al., 2025). At the molecular level, curcumin dose-dependently reduces intracellular ROS, inhibits mitochondrial membrane potential collapse and cytochrome c release, and suppresses caspase-9/caspase-3 activation, thereby interrupting mitochondria-dependent apoptosis (Liu et al., 2011). Additionally, by inhibiting the hyperphosphorylation of c-Jun N-terminal kinase, curcumin prevents the mitochondrial translocation of the pro-apoptotic factor Bax, subsequently reducing dopaminergic neuronal death and striatal axonal degeneration (Pan et al., 2012). The coordination polymers, composed of ferric ions and curcumin at an ultrasmall nanoscale, exhibit blood-brain barrier permeability, which can effectively scavenge free radicals, inhibit neuroinflammation and restore mitochondrial function. These polymers significantly alleviate oxidative stress and inflammatory states in the midbrain and striatum (Cheng et al., 2022). In animal models, curcumin treatment (80 mg/kg) reversed rotenone (2.5 mg/kg)-induced motor coordination deficits while restoring antioxidant capacity and mitochondrial function. The rotenone-induced PD model displayed compromised autophagy, characterized by reduced p62 and LC3-II expression levels which led to excessive accumulation of misfolded α-syn. In contrast, curcumin promoted the autophagic clearance of pathological α-syn by upregulating LC3-II expression and inhibiting the apoptotic cascade. Furthermore, curcumin administration upregulated Nrf2 expression and normalized the Nrf2-Keap1 pathway, thereby enhancing antioxidant activity (Rathore et al., 2023). The curcumin analogue-derived nanoscavenger (NanoCA) with a controlled-release property was designed to facilitate the nuclear translocation of TFEB, which activated both autophagy and calcium-dependent exosome secretion to clear α-syn. Pre-treatment with NanoCA has been shown to shield cell lines and primary neurons from MPP^+^-induced neurotoxicity. Significantly, a novel rapid arousal intranasal delivery system (RA-IDDS) was developed to ensure targeted delivery of NanoCA to the brain, which provided substantial neuroprotection to alleviate behavioral deficits and enhance the clearance of monomers, oligomers and aggregates of α-syn in the midbrain (Liu et al., 2020). Emerging evidence suggests that the activation of the gut-brain axis through intestinal inflammation may contribute to PD pathogenesis, with gastrointestinal dysfunction representing the most common non-motor symptom (Kotsiliti, 2023). In the N-methyl-4-phenyl-1,2,3,6-tetrahydropyridine (MPTP)-induced PD mouse model, curcumin intervention attenuated the reduction of tyrosine hydroxylase levels in both substantia nigra pars compacta and striatum, inhibited AIM2-mediated pyroptosis and caspase-1 activation, and decreased pro-inflammatory factors and inflammatory responses in the intestinal tract. The gastrointestinal disorders and barrier function were improved, and motor dysfunction and dopaminergic neuron loss were reversed (Zhong et al., 2022; Cai et al., 2023). These findings highlight curcumin’s potential as a multi-target therapeutic agent in PD, addressing both central and peripheral mechanisms of the disease.

### 4.3 Amyotrophic lateral sclerosis

#### 4.3.1 Pathogenesis of ALS

Amyotrophic lateral sclerosis (ALS) is pathologically defined by the degeneration of both upper and lower motor neurons followed by muscle denervation, atrophy and ultimately profound motor disability. The annual incidence is 1.5–2.7 per 100,000 individuals, with a corresponding prevalence of 3-5 per 100,000 (Xu L. et al., 2020). The peak period of disease onset is between 55 and 65 years old. Approximately 10% of ALS patients are hereditary, called familial ALS (fALS), while the remaining 90% (sALS) occur sporadically without apparent genetic predisposition (Van Daele et al., 2023). Notably, the mean onset age of fALS tends to be earlier than that of sALS. Cognitive impairments occur in 20%–50% of cases, with a subset (5%–15%) progressing to full-blown frontotemporal dementia. The average survival time ranges from 2 to 5 years post-diagnosis, and respiratory failure is the primary cause of mortality. About 5%–10% of individuals exhibit significantly prolonged survival exceeding 10 years (Su W. et al., 2021). The exact pathogenic mechanisms of ALS have not been fully elucidated. Potential risk factors include genetic predisposition, neurotoxicant exposure (organic solvents, pesticides and heavy metals), chronic nutritional deficiencies, metabolic disorders, immune dysregulation, viral infections and neuroinflammation (Newell et al., 2022; Xue et al., 2022). The widely accepted hypothesis proposes that motor neuron degeneration arises from the interaction between genetic susceptibility and environmental factors, primarily through mitochondrial dysfunction and cytoskeletal disruption mediated by oxidative stress and excitotoxicity (Zhou et al., 2023). The neurodegenerative cascade typically originates at the neuromuscular junction and distal axons, characterized by early synaptic dysfunction and axonal degeneration that progresses to neuronal death, ultimately causing neural transmission failure and motor impairment (Berth and Lloyd, 2023). Axonal lesions are the earliest clinical symptom and crucial cause of ALS (Luan et al., 2024). Among the ALS-associated genes, multiple mutations have been demonstrated to be strongly correlated with axonal dysfunction (Mizielinska et al., 2025). The highly efficient transport system within neuronal axons is essential for maintaining material and energy supply, and transport defects constitutes a major contributor to ALS. Kinesin, a motor protein responsible for anterograde transport of organelles, vesicles, and protein complexes from the cell body to synaptic terminals, causes transport disruption when mutated. Conversely, the dynein mediates retrograde transport of various cargoes, and ALS-related mutations lead to transport abnormalities, axonal swelling and ultimately motor neuron degeneration (Cristofani et al., 2017). Additionally, the change of tubulin can destabilize microtubule networks, which also impairs axonal transport (Fumagalli et al., 2021).

The abnormal accumulation of proteins in motor neurons is a hallmark pathological feature of ALS, mainly including transactive response DNA-binding protein 43 (TDP-43), fused in sarcoma (FUS), SOD1 and sequestosome 1 (SQSTM1). RNA-binding proteins, particularly TDP-43, play a key role in protein aggregation. TDP-43 aggregates are found in over 90% of ALS cases (Lepine et al., 2022). Most disease-causing mutations in these proteins occur in low-complexity domains mediating liquid-liquid phase separation to alter protein conformation, which promotes the aggregation and disrupts RNA regulation while also generating additional toxicity. Notably, many protein aggregates also present in neuronal axons, such as mutated TDP-43 and FUS, resulting in abnormal RNA transport and dysregulated protein synthesis (Marrone et al., 2019). *SOD1* was the first identified ALS-associated gene, and mutations alter the protein conformation to expose hydrophobic surfaces and facilitate the aggregation (Wang L. et al., 2024). C9ORF72 (chromosome 9 open reading frame 72) is another core genetic contributor to ALS. The expansion of a G4C2 (GGGGCC) hexanucleotide repeat in the non-coding region leads to the production and accumulation of dipeptide repeat proteins in neurons (Maor-Nof et al., 2021). In addition, SOD1, C9ORF72, TDP-43 and FUS are also crucial molecular players associated with mitochondrial defects in ALS. The SOD1 G93A mutation results in its accumulation within axons and significantly impedes mitochondrial trafficking. Furthermore, SOD1 G93A may form pathogenic complexes with Bcl-2 to potentiate cytochrome c release and subsequent activation of apoptotic pathways (Pedrini et al., 2010). Other SOD1 mutants, including A4V, H46R, D90A and R115G, have been also found to disrupt proteostasis, diminish mitochondrial membrane potential and compromise ATP generation (Rasouli et al., 2018). The poly-dipeptides derived from C9ORF72 hexanucleotide expansions exhibit high affinity for mitochondrial proteins, thereby impairing mitochondrial structural integrity and intracellular transport dynamics, as well as Ca^2+^ uptake and ATP synthesis (Fumagalli et al., 2021). Pathogenic mutations in TDP-43, like Q331K and M337V, interfere with the microtubule-dependent mitochondrial transport system, leading to profound mitochondrial dysfunction. Excessive TDP-43 can also induce mitochondrial DNA leakage into the cytosol, which activates NF-κB and type I interferon pathways that drive neuroinflammatory responses (Yu et al., 2020). In summary, aberrant protein changes are crucial of ALS. Nevertheless, the distinct aggregation behaviors underscore the multifaceted nature of the pathology and present formidable challenges for therapeutic development.

#### 4.3.2 Function of curcumin in ALS

Curcumin is recommended as a dietary supplement to reduce the risk of ALS (D'Antona et al., 2021). However, to date, research on its therapeutic potential in ALS remains limited (de Oliveira et al., 2016). Both mutant and wild-type TDP-43 elevate neuronal excitability, with mutant TDP-43 exhibiting greater toxicity. The hyperexcitability is accompanied by oxidative stress and mitochondrial dysfunction. Treatment with dimethoxy curcumin (DMC) significantly normalizes aberrant action potentials and voltage-gated sodium (Nav) channel activity, indicating a dropping-excitability state. The rescue involves DMC-mediated mitigation of oxidative stress and restoration of mitochondrial function (Dong et al., 2014). Men and postmenopausal women show a higher incidence and more severe progression of ALS and FTLD (frontotemporal lobar degeneration) compared to younger women, pointing to a key role for sex hormones in disease development (McCombe and Henderson, 2010). In Prp-TDP-43^A315T^ mice, dysregulated expression of estrogen-related enzymes (CYP19A1 and CYP3A4) was observed relative to wild-type controls, which suggested that toxic phosphorylated TDP-43 oligomers may disrupt the balance between CYP19A1 and CYP3A4 expression, resulting in reduced estrogen biosynthesis and accelerated degradation. Remarkably, oral administration of solid lipid curcumin particles (SLCP) improved survival in female Prp-TDP-43^A315T^ mice and diminished pathological TDP-43 deposits. The treatment also mitigated TDP-43-driven neurodegeneration by regulating estrogen production and CYP450 enzyme activity. These findings highlight SLCP as a potential estrogen replacement therapy for ALS and FTLD (Majumder et al., 2025). Curcumin shows enhanced binding affinity for mutant proteins compared to the native form, primarily through increased hydrophobic interactions (Srinivasan and Rajasekaran, 2016). By targeting amyloidogenic regions, curcumin blocks SOD1 fibrillation and promotes the formation of smaller and disordered aggregates. While pre-fibrillar and fibrillar SOD1 aggregates exhibit significant toxicity, this effect is absent in curcumin-treated samples, suggesting its ability to neutralize harmful intermediates (Bhatia et al., 2015). Additionally, curcumin potently inhibits FUS liquid-liquid phase separation by suppresses droplet formation and stress granule assembly. Based on hydrophobic interactions, it reduces FUS β-sheet content, mitigates aberrant aggregation, and rescues cellular metabolic dysfunction by reversing FUS-induced sequestration of pyruvate kinase and restoring ATP production. (Shi et al., 2023). These properties position curcumin as a promising candidate for ALS, but further investigation is still warranted to fully elucidate its efficacy and mechanism of action.

### 4.4 Huntington’s disease

#### 4.4.1 Pathogenesis of HD

Huntington’s disease (HD) is an autosomal dominant neurodegenerative disorder characterized by progressive motor dysfunction, cognitive decline and psychiatric disturbances, which typically manifests in mid-adulthood with an average age of 30–50 years old (Ling et al., 2025; Eddy et al., 2016). It has been reported across diverse ethnic populations, with the highest prevalence observed in Caucasian populations (10.6–13.7/100,000). In contrast, Asian populations including those in Japan, Taiwan (China) and Hong Kong (China) demonstrate substantially lower prevalence (0.1–0.7/100,000), suggesting potential genetic and/or environmental modifying factors (Wise, 2010; Buchanan et al., 2023). The disease is invariably fatal, with a life expectancy of 15–20 years after symptom onset. The neuropathological features of HD are selective and massive degeneration of medium spiny neurons (MSNs) in the striatum, and mild degeneration of cortical pyramidal neurons, accompanied by astrocyte proliferation (Wright et al., 2020). It arises from an abnormally expanded CAG trinucleotide repeat encoding the elongated polyglutamine (polyQ) within exon 1 of *Huntingtin* (*HTT*) gene, which results in the production of toxic mutant huntingtin proteins (mHTT) that accumulate in neurons, particularly in the striatum and cortex (Genetic Modifiers of Huntington’s Disease Consortium, 2019). In healthy individuals, the *HTT* gene normally contains 10 to 35 CAG trinucleotide repeats, while HD patients exhibit more than 36 repeats. Emerging evidence indicates that pronounced neurotoxic effects manifest only when CAG repeats exceed 150 copies. These exceptionally large repeat expansions demonstrate striking cellular specificity, being predominantly localized to MSNs (Matlik et al., 2024). Upon exceeding 150 CAG repeats, MSNs undergo progressive loss of neuronal characteristics while concurrently upregulating senescence-related and pro-apoptotic gene programs, culminating in neuronal degeneration. Notably, MSNs maintain stable gene expression profiles when CAG repeat lengths remain below the critical threshold (Handsaker et al., 2025). Longer CAG repeats are correlated significantly with earlier onset age and more severe clinical manifestations (Genetic Modifiers of Huntington’s Disease Consortium, 2019).

Genome-wide association studies have identified potential genetic modifiers, including mismatch-repair (MMR) genes (*MSH3*, *MLH1*, *PMS1* and *PMS2*) and other DNA repair genes (*FAN1* and *LIG1*) (Aldous et al., 2024). The MMR system comprises specialized protein complexes with distinct functions: MutSβ (MSH2-MSH3 heterodimer) primarily recognizes large insertion-deletion loops (IDLs) and repetitive sequence extrusions, whereas MutSα (MSH2-MSH6 heterodimer) detects smaller 1-2 bp IDLs (Ferguson et al., 2024). Following mismatch recognition, MutS complexes recruit specific MutL heterodimers including MutLα (MLH1-PMS2), MutLβ (MLH1-PMS1) and MutLγ (MLH1-MLH3) (Dai et al., 2021; Ortega et al., 2021). While MutLα and MutLγ possess critical endonuclease activities essential for MMR initiation, the biological role of MutLβ remains enigmatic due to the lack of demonstrated nuclease activity. The knockout of MMR genes (*MSH3*, *PMS1*, *MSH2* and *MLH1*) ameliorates both early-onset phenotypes in MSNs and late-onset manifestations in cortical neurons, including somatic CAG-repeat expansion, transcriptionopathy and mHTT aggregation (Moss et al., 2017). *MSH3* deficiency specifically improves open-chromatin dysregulation in neurons. The rapid linear expansion rate of CAG repeats in MSNs is sharply reduced or terminated by MMR mutants. The deficiencies in either *MSH3* or *PMS1* effectively prevent mHTT aggregation by maintaining somatic CAG repeat lengths below the critical threshold of 150 repeats (Wang N. et al., 2025). Moreover, *MSH3* deficiency demonstrates comprehensive therapeutic potential by correcting synaptic dysfunction, astrocytic abnormalities and locomotor deficits in HD mice (Bunting et al., 2025). These findings establish that *MSH3* and *PMS1* play pivotal roles in driving accelerated somatic mHTT CAG expansion rates specifically in HD-vulnerable neurons, underlying the repeat length-dependent, threshold-sensitive, selective and progressive pathogenesis *in vivo*.

TDP-43 is a DNA/RNA-binding protein essential for splicing regulation. TDP-43 and the N6-methyladenosine (m6A) writer protein METTL3 are upstream regulators of exon skipping in HD (Mendel et al., 2018). TDP-43 exhibits disrupted nuclear localization and accumulates in the cytoplasm in a phosphorylated form in HD mouse and human brains. Notably, TDP-43 co-localizes with mHTT nuclear aggregates, suggesting a potential interaction within pathological inclusion bodies (Sanchez et al., 2021). The binding of TDP-43 to RNAs encoding HD-associated genes, many of which are differentially expressed and aberrantly spliced, is significantly reduced. Concurrently, m6A RNA modifications are diminished on RNAs that are abnormally expressed in the striatum, particularly at clustered sites near TDP-43 binding regions (Yu et al., 2018). The spatial and functional overlap between reduced m6A marks and impaired TDP-43-RNA interactions suggests a synergistic mechanism: the loss of METTL3-mediated m6A modification may destabilize RNA-protein complexes, exacerbating the inability of TDP-43 to regulate splicing fidelity (Tollervey et al., 2011). These findings support TDP-43 dysfunction and altered m6A modification converge to drive alternative splicing defects in HD (Nguyen et al., 2025). It has been also unveiled that the adenosine can be methylated to N^1^-methyladenosine (m1A) by the enzyme TRMT61A, while m1A can be demethylated by ALKBH3. The m1A/adenosine ratio in CAG repeat RNA exhibits a positive correlation with repeat length, a phenomenon attributed to the reduced expression of ALKBH3 elicited by the repeat RNA. TDP-43 binds directly and with high affinity to m1A in RNA, which promotes the cytoplasmic mis-localization and subsequent formation of gel-like aggregates (Sun et al., 2023).

#### 4.4.2 Function of curcumin in HD

Although multiple cellular events, including oxidative stress, mitochondrial dysfunction, neuroinflammation and transcriptional dysregulation, have been identified in HD, the development of effective therapies with favorable safety profiles remains an unmet medical need (Elifani et al., 2016). Curcumin demonstrated potent anti-aggregation effects in yeast, significantly inhibiting the formation of both htt72Q-GFP (a polyglutamine-rich protein) and Het-s-GFP (a non-polyglutamine protein) aggregates. It was found that curcumin prevented htt72Q-GFP aggregation through the downregulation of Vps36, a crucial component of the ESCRT-II complex. On the other hand, it also exhibited disaggregation activity, effectively disrupting pre-formed htt72Q-GFP aggregates (Verma et al., 2012). Curcumin treatment enhances mitochondrial function by upregulating the activity of electron transport chain complexes and elevating cytochrome levels. This restoration is accompanied by increased glutathione content and SOD activity, indicating improved redox homeostasis. Furthermore, curcumin administration significantly attenuates mitochondrial swelling, reduces lipid peroxidation and protein carbonyl formation, and decreases ROS production in HD rats (Sandhir et al., 2014). These protective effects are mediated partly through the activation of Nrf2 antioxidant pathway, leading to marked improvements in neuromotor coordination (Santana-Martínez et al., 2019). The CAG140 knock-in (KI) mouse model exhibits abnormal mHTT aggregates, striatal transcriptional deficits, and early-onset motor, cognitive and affective impairments. Curcumin-supplemented KI mice showed reduced mHTT aggregation and upregulated mRNA expression of striatal DARPP-32 and dopamine receptor D1, which were accompanied by behavioral recovery, particularly in rearing deficits (Hickey et al., 2012). Curcumin enhanced the phagocytic activity by upregulating the expression of key phagocytic receptors in HD flies, while simultaneously suppressing the transcriptional expression of pro-inflammatory cytokines and anti-microbial peptides (Dhankhar et al., 2023). It was found that curcumin normalized body weight and restored lipid and carbohydrate homeostasis. The treatment also reduced elevated ROS levels in adipose tissue and significantly improved both survival rates and locomotor function, even at advanced disease stages (Aditi et al., 2022). A recent study elucidated the mechanism of curcumin modulating the aggregation of huntingtin exon 1. Under substoichiometric amounts, curcumin was found to perturb primary and/or secondary nucleation events, thereby extending the lag phase preceding aggregation. Notably, this disruption of the aggregation kinetics not only altered the structural properties of the resulting aggregates but also their cellular metabolic profiles. When applied to neuronal cells, the “break-through” aggregates, formed in the presence of curcumin, induced markedly lower cellular stress compared to those generated without the inhibitor. It was believed that the differential effect was attributed to structural modifications, particularly the presence or absence of the polyglutamine (polyQ) β-hairpin conformation in the aggregates (Jain et al., 2025).

## 5 Strategies to enhance the bioavailability and pharmacological efficacy of curcumin

### 5.1 Poor bioavailability of curcumin

Although curcumin has demonstrated excellent pharmacological activities, the extremely low bioavailability significantly restricts its clinical application in disease prevention and treatment. After a single oral dose of 10 or 12 g of curcumin for 0.25–72 h, almost no free form is detectable in human plasma (Vareed et al., 2008). Achieving health-promoting effects often requires long-term intake of high doses of curcumin, which may lead to adverse reactions and poor compliance. The low bioavailability is attributed to multiple factors, including poor solubility, low stability, inefficient absorption and rapid metabolism and excretion (Sabet et al., 2021). Curcumin exhibits poor stability in aqueous solutions and degrades rapidly in a pH-dependent manner. Its half-life ranges from approximately 100–200 min at pH 3–6.5, but sharply decreases to 1–9 min at pH 7.2–8.0. Notably, the biological activity of the hydrolysis products is generally lower or negligible. The primary absorption site of curcumin in the gastrointestinal tract (GIT) is the small intestine (Luo et al., 2022). As a lipophilic polyphenolic compound, curcumin is difficult to be absorbed by small intestinal epithelial cells. Even when absorbed, its hydrophobic nature often leads to efflux back into the intestinal lumen via the P-glycoprotein transporter, which is highly expressed in the intestine (Culbertson and Liao, 2025). This process significantly reduces curcumin’s entry into the bloodstream. Following oral administration, curcumin undergoes rapid metabolism, including reduction (Phase I metabolism) and conjugation (Phase II metabolism) in GIT and liver. Enzymes like NADPH reductase metabolize curcumin into various products, including dihydrocurcumin, tetrahydrocurcumin, hexahydrocurcumin and octahydrocurcumin (Metzler et al., 2013). Additionally, gut bacteria, like *Escherichia coli*, can catalyze curcumin reduction through a unique bacterial enzyme, CurA (NADPH-dependent curcumin/dihydrocurcumin reductase), resulting in the production of dihydrocurcumin and tetrahydrocurcumin (Hassaninasab et al., 2011). The two phenolic groups of curcumin also make it susceptible to Phase II detoxifying enzymes, such as glucuronosyltransferases and sulfotransferases, leading to the formation of glucuronide and sulfate conjugates (Pan et al., 1999). These metabolites possess significantly reduced biological activity compared to curcumin itself. Conventional tablet or powder formulations release curcumin quickly in the GIT but fail to address the poor solubility and metabolic challenges. Non-oral routes, such as intravenous injection, can bypass some of these issues; however, curcumin tends to aggregate or precipitate in the bloodstream, raising safety concerns. Therefore, improving curcumin’s bioavailability remains a critical challenge for its therapeutic potential.

### 5.2 Nano-delivery systems

To overcome the above limitations, several strategies have been developed to enhance the utilization efficiency and pharmacological efficacy of curcumin, focusing on nanoparticle formulations, chemical modification, novel delivery systems and drug combination. Particle size is closely related to drug solubility. Nanoparticle-based formulations, including nanoemulsions, micelles, liposomes and solid dispersions, are widely used to improve curcumin’s solubility and bioavailability (Xu et al., 2024; Olmos-Juste et al., 2021). Through non-covalent interactions, curcumin (Cur) and poly (−)-epigallocatechin-3-gallate (pEGCG) were co-encapsulated within hyaluronic acid (HA) to form HA-CurNPs nanoparticles that exhibited sustained-release properties (Chen T. et al., 2024). The released drugs synergistically scavenged ROS and inhibited apoptosis while modulating CD74 to promote microglial polarization toward the M2 phenotype, thereby alleviating neuroinflammation and enhancing neurogenesis. Additionally, these nanoparticles effectively maintained neuronal integrity, reduced glial scarring, and ultimately restored electrical signaling and motor functions at injury sites. To overcome the blood-brain barrier, curcumin-loaded polysorbate 80-modified cerasome nanoparticles (CPC NPs, ∼110 nm) were developed, coupled with ultrasound-targeted microbubble destruction (UTMD) to enhance brain targeting (Zhang et al., 2018). The nanohybrid cerasomes demonstrated superior stability against surfactant solubilization and extended circulation time. The combined CPC NPs-UTMD approach achieved 1.7-fold greater brain permeation compared to CPC alone at 6 h post-administration. In MPTP-induced PD models, this strategy effectively delivered curcumin to the striatum, clearing α-syn aggregates and restoring normal motor function, dopamine levels and tyrosine hydroxylase expression. This novel delivery system with higher therapeutic relevance and fewer unwanted complications could be a promising choice for PD treatment. Liposomes, composed of phospholipid bilayers, can encapsulate curcumin, enhancing its hydrophilicity and mimicking cell membranes to promote transmembrane absorption (Fernandes et al., 2021). PEGylation of liposomes further reduces the clearance of the reticuloendothelial system and prolongs systemic circulation. Solid dispersions, such as those prepared with polyvinylpyrrolidone or poloxamer, increased curcumin’s solubility and stability by converting crystalline forms into amorphous structures (Yang et al., 2023). However, low encapsulation efficiency, complex preparation processes and instability persist as challenges in nanoparticle-based systems.

### 5.3 Chemical structure modification

Chemical structure modification, including synthesizing analogs and designing prodrugs, is another effective approach. Curcumin predominantly exists in the bis-keto form in acidic to neutral conditions (pH 3–7), stabilized by an acidic proton on the activated interaromatic carbon. Conversely, under alkaline conditions (pH > 8), the enolic form becomes predominant, conferring electron-donating properties (Genchi et al., 2024). The β-diketone moiety, responsible for keto-enol tautomerism, can be modified to enhance its antioxidant activity. 3,5-bis(2-fluorobenzylidene)piperidin-4-one (EF24) demonstrated potent NF-κB suppression by directly inhibiting IKK (Kasinski et al., 2008). EF24 blocked TNF-α-induced IκB phosphorylation and degradation, with *in vitro* studies confirming the inhibition of IKK catalytic activity. These findings identified IKK as the molecular target of EF24 and elucidated its superior efficacy compared to curcumin prototype. Prodrugs are pharmacologically inactive derivatives that are metabolized into active forms within the body. Common modifications involve esterification, etherification, hydrazone and disulfide bond formation. For example, curcumin linked to glutaric acid via an ester bond showed improved water solubility, while curcumin conjugated with poly (lactic-co-glycolic acid) enabled controlled release, enhancing cellular uptake (Muangnoi et al., 2018; Sampath et al., 2014). In addition, co-loading curcumin with doxorubicin in pH-sensitive nanoparticles has demonstrated enhanced bioavailability and reduced systemic toxicity (Zhang et al., 2016). Some prodrugs require specific enzymes or endogenous compounds for hydrolysis, such as the carboxylesterases including acetylcholinesterase, butyrylcholinesterase and arylesterase that are critically involved in the bioconversion of ester-based prodrugs into pharmacologically active forms (Liederer and Borchardt, 2006). Due to the substrate specificity of different esterases, the enzymatic consideration should be systematically incorporated into the early design stage.

### 5.4 Combination drug therapy

Combination therapies aim to improve curcumin bioavailability by inhibiting its inactivation and metabolism. Curcumin is primarily metabolized by UDP-glucuronosyltransferases and sulfotransferases. Natural compounds such as piperine, quercetin and silymarin have been shown to inhibit curcumin glucuronidation, thereby increasing its plasma levels and oral bioavailability (Grill et al., 2014; Rinwa and Kumar, 2013). Synergistic effects have also been observed when curcumin is combined with other bioactive compounds-naringenin, genistein and epigallocatechin gallate (Ghobadi and Asoodeh, 2023). For the rats administered either curcumin (25 or 50 mg/kg) or a combination of curcumin (25 mg/kg) with piperine (2.5 mg/kg) once daily for 21 consecutive days prior to 3-Nitropropionic acid administration (3-NP, a neurotoxin inducing symptoms of Huntington’s disease), it was found that both curcumin monotherapy and the curcumin-piperine combination demonstrated protective effects against 3-NP-induced motor deficit, biochemical alterations and neurochemical abnormalities. The combination treatment showed greater neuroprotection compared to curcumin alone, suggesting piperine as a bioavailability enhancer of curcumin (Singh et al., 2015). In the clinical trial involving 48 AD patients, there was no remarkable differences between the unformulated standard curcumin treatment and the placebo group. However, the curcumin-galactomannan complex significantly improved the MMSE and GLFS scores of the participants as well as the serum levels of specific biomarkers (Das et al., 2023).

## 6 Conclusion and prospect

### 6.1 Conclusion

Neurodegenerative diseases represent a significant threat to public health, exacerbated by the global aging population. The pathogenesis of NDs, such as AD, PD, HD and ALS, is complex and multifactorial, involving oxidative stress, neuroinflammation, mitochondrial dysfunction and protein misfolding/aggregation. These pathological processes lead to progressive neuronal loss and subsequent neurological dysfunction. Curcumin, a natural polyphenolic compound, has emerged as a promising therapeutic candidate for NDs due to its excellent biological properties, including potent antioxidant, anti-inflammatory, anti-apoptotic and neuroprotective effects. It has been shown to scavenge ROS, inhibit pro-inflammatory pathways, reduce the accumulation and toxicity of misfolded proteins, and promote neuronal survival and regeneration. To overcome the limitations of curcumin’s low bioavailability in clinical applications, various strategies have also been developed to enhance the utilization efficiency and pharmacological efficacy, including nano-delivery systems, chemical modifications and combination therapies. *In vitro* experiments and a small number of clinical studies have confirmed the role of curcumin-related preparations in alleviating neurodegeneration and delaying disease progression. This approach is considered an additional option for managing the health issues arising from the global demographic shift toward an aging population.

### 6.2 Future prospect

Although curcumin has demonstrated potential neuroprotective effects, it is important to highlight that the clinical transformation still encounters numerous challenges and requires further investigation: (1) Most curcumin formulations used in current studies are complex mixtures primarily consisting of curcumin, demethoxycurcumin and bisdemethoxycurcumin. Moreover, significant variations exist across studies regarding the source, purity, and formulation of curcumin, complicating the comparison of clinical outcomes. To address this issue, standardized quality control protocols for curcumin preparations should be established to minimize batch-to-batch inconsistencies. (2) Clinical evidence remains inconsistent, with some trials reporting limited efficacy, potentially due to inadequate dosing, suboptimal trial design (e.g., insufficient sample size or duration), or patient heterogeneity. Additionally, high doses may induce gastrointestinal discomfort, and the potential hepatorenal toxicity necessitates further evaluation. Large-scale and long-term clinical trials are essential to resolve these concerns. (3) While curcumin exhibits a broad spectrum of molecular targets, its pleiotropic effects complicate the elucidation of precise mechanisms of action, thereby hindering targeted therapeutic applications. (4) Despite various strategies proposed to enhance curcumin’s bioavailability and therapeutic efficacy, the *in vitro*-*in vivo* correlation of cell cytotoxicity and bioavailability remains poorly understood, warranting additional human studies. (5) A comprehensive assessment of the safety, stability and long-term effects of excipients and polymers utilized in novel delivery systems is required. Current *in vitro* and animal models inadequately replicate the complexity of human gastrointestinal absorption, limiting their predictive value for drug behavior in humans. Further research into pharmacokinetics, pharmacodynamics and optimal delivery strategies is necessary for successful clinical application. (6) Integrating curcumin with complementary therapies, such as lifestyle modifications, dietary interventions and physical activity, holds promise for improving outcomes in NDs. Incorporating curcumin into comprehensive treatment regimens could potentially slow ND progression and enhance life quality, addressing the growing global health challenge.
